# A Robust Handwritten Numeral Recognition Using Hybrid Orthogonal Polynomials and Moments

**DOI:** 10.3390/s21061999

**Published:** 2021-03-12

**Authors:** Sadiq H. Abdulhussain, Basheera M. Mahmmod, Marwah Abdulrazzaq Naser, Muntadher Qasim Alsabah, Roslizah Ali, S. A. R. Al-Haddad

**Affiliations:** 1 Department of Computer Engineering, University of Baghdad, Al-Jadriya 10071, Iraq; sadiqhabeeb@coeng.uobaghdad.edu.iq (S.H.A.); basheera.m@coeng.uobaghdad.edu.iq (B.M.M.); 2 Continuous Education Center, University of Baghdad, Baghdad 10001, Iraq; marwah@dcec.uobaghdad.edu.iq; 3 Department of Electronic and Electrical Engineering, University of Sheffield, Sheffield S1 4ET, UK; mqalsabah1@sheffield.ac.uk; 4 Department of Computer and Communication Systems Engineering, Faculty of Engineering, Universiti Putra Malaysia, Serdang 43400, Malaysia; roslizah@upm.edu.my

**Keywords:** character recognition, orthogonal polynomials, orthogonal moments, Krawtchouk polynomials, Tchebichef polynomials, support vector machine

## Abstract

Numeral recognition is considered an essential preliminary step for optical character recognition, document understanding, and others. Although several handwritten numeral recognition algorithms have been proposed so far, achieving adequate recognition accuracy and execution time remain challenging to date. In particular, recognition accuracy depends on the features extraction mechanism. As such, a fast and robust numeral recognition method is essential, which meets the desired accuracy by extracting the features efficiently while maintaining fast implementation time. Furthermore, to date most of the existing studies are focused on evaluating their methods based on clean environments, thus limiting understanding of their potential application in more realistic noise environments. Therefore, finding a feasible and accurate handwritten numeral recognition method that is accurate in the more practical noisy environment is crucial. To this end, this paper proposes a new scheme for handwritten numeral recognition using Hybrid orthogonal polynomials. Gradient and smoothed features are extracted using the hybrid orthogonal polynomial. To reduce the complexity of feature extraction, the embedded image kernel technique has been adopted. In addition, support vector machine is used to classify the extracted features for the different numerals. The proposed scheme is evaluated under three different numeral recognition datasets: Roman, Arabic, and Devanagari. We compare the accuracy of the proposed numeral recognition method with the accuracy achieved by the state-of-the-art recognition methods. In addition, we compare the proposed method with the most updated method of a convolutional neural network. The results show that the proposed method achieves almost the highest recognition accuracy in comparison with the existing recognition methods in all the scenarios considered. Importantly, the results demonstrate that the proposed method is robust against the noise distortion and outperforms the convolutional neural network considerably, which signifies the feasibility and the effectiveness of the proposed approach in comparison to the state-of-the-art recognition methods under both clean noise and more realistic noise environments.

## 1. Introduction

Due to the advancement of computational capabilities of computers, the automatic processing of commercial applications that deals with handwriting has gained substantial attention around the world. In particular, handwriting digit recognition plays a vital role in the area of pattern recognition and computer vision, which are considered as an essential subfield of optical character recognition (OCR) [1]. Both handwritten and printed numerals recognition have gained considerable attention in different practical applications. Specifically, handwritten numeral recognition can be used for several purposes: (a) processing cheques in the bank sector, (b) scanning zip code in the post offices, (c) recognition of vehicle number, (d) processing medical data, and (e) recognition of the street number [1,2,3,4]. However, automatic recognition of handwritten numeral is to date considered to be a challenging issue. This is due to the huge variations in handwriting styles, which imply that the same numeral can be written in various ways depending on the font size, font orientation, and the usage of different writing materials. Therefore, to address these challenges, developing an accurate handwritten numeral recognition scheme is crucial. In addition, the trade-off between the process of extracting the most informative features, which have the ability to enhance the classification accuracy, and the complexity reduction is to date one of the most critical issue in this area of research. Furthermore, handwritten numeral recognition is defined as the process of recognizing and classifying numerals from 0 to 9 without any human involvement [5]. While extensive research works have been carried out for the recognition of handwritten numerals and several different techniques have already been proposed, improving the recognition accuracy rates are still required [6].

## 2. Related Works

In [7], different feature extraction classifiers have been developed using cooperation of four support vector machine (SVM) for Roman handwritten digit recognition. To this end, four different types of feature extractions, namely, projection histograms, ring-zones, contour profiles, and Kirsch features, have been investigated. Chen et al. [8] examined a max-min pseudo-probabilities approach for Roman handwritten numerals recognition. In this study, 256 different dimensions features have been extracted from an input image through the use of principal component analysis (PCA). Note that the research works in [7,8] have been carried out using the National Institute of Standards and Technology (NIST) digital database in [9]. Shi et al. [10] proposed an algorithm using gradient and curvature features. These features are fused to compose a feature vector to classify Roman numerals. The work in [11] examined a feature extraction for handwritten numeral recognition using a sparse coding strategy with a local maximum operation. This method has been evaluated using a modified NIST (MNIST) digital database in [12]. In [13], a handwritten numeral recognition approach, which utilizes multiple feature extraction methods and classifier ensembles, has been proposed. In this proposed scheme, six different features of extraction methods have been evaluated. In [6], a moment-based approach has been proposed for handwritten digit recognition. In particular, the proposed scheme has been evaluated under several scripts: (a) Indo-Arabic, (b) Bangla, (c) Devanagari, (d) Roman, and (e) Telugu. The proposed method in [6] has been examined using the databases from the Center for Microprocessor Applications for Training Education and Research (CMATER) and MNIST.

Besides the research studies that used feature extraction approaches for handwritten digit recognition, another line of research studies has focused on using the neural network (NN) method for numeral recognition. To this end, several NN-based architectures have been proposed for numeral recognition. For example, in [14,15] a trainable feature extraction has been introduced based on convolutional neural network architecture (CNN) with SVM based classifier. The proposed scheme has been applied on the MNIST database. In [16], a new limited receptive area (LiRA) features has been investigated for the handwritten Roman recognition based on MNIST database, which has been developed in [17]. Subsequently, two different NN classifiers have been considered: (a) a modified 3-layer perceptron LiRA and (b) a modular assembly neural network. A new method based on Boltzmann machine (RBM) and CNN deep learning method has been proposed in [18] for Arabic handwritten digit recognition. The proposed method has been applied in the CMATERDB 3.3.1 Arabic handwritten digit datasets. In [19], a CNN algorithm has been proposed for the Arabic numeral recognition, which uses several convolutional layers along with ReLU activation. In [20], an offline handwritten recognition based on DNN has been developed for both digits and letters. The proposed method has been examined using the MNIST and EMNIST databases. Several studies have investigated the enhancement of the digits misrecognitions by adopting the deep learning-based deep belief network (DBN), see, e.g., in [21,22]. However, the DBN methods suffer from a poor recognition accuracy and high running time, which make such methods unfeasible for practical implementation. To overcome these issues, a combined method based on feature extraction and decision making of reinforcement learning has been proposed, see, e.g., in [23,24]. In particular, an adaptive deep Q-learning method has been proposed in [3] aiming to enhance the recognition accuracy and reducing the running time for handwritten digit recognition. In [1], two CNN-based approaches have been investigated for handwritten Arabic digit recognition. In the research, a new development in the size of the Arabic numerals database from 3,000 to 72,000 has also been introduced. Both the CNN models have achieved a recognition accuracy that is close to the state-of-the-art methods for Arabic numerals recognition.

Two shape-based feature descriptors, which are termed as a Point-Light Source-based Shadow (PLSS) and Histogram of Oriented Pixel Positions (HOPP), have been proposed in [25]. The proposed framework has been implemented using ten of the available datasets of handwritten digits, which are written in eight different languages and one numeric string recognition dataset. The nine offline handwritten digits datasets include Bangla, Arabic, Telugu, Nepali, Assamese, two versions of Gurumukhi Latin and two versions of Devanagari, and one online Assamese numeral dataset.

In [26], a hybrid of PCA and modular PCA (MPCA) recognition method with quad-tree-based hierarchically derived longest-run (QTLR) features has been proposed for optical character recognition of handwritten digits using SVM classifier. In this study, five popular Indian sub-continent scripts have been evaluated, which include Arabic, Bangla, Devanagari, Latin, and Telugu. The authors of [27] have proposed the 5-layer CNN method with SVM classifier for efficient handwritten recognition. In this work, 50-class BangIa datasets samples have been used in the training. In particular, this method has extracted the features of five different 10-class problems of Indian scripts, which are English, Devanagari, BangIa, Telugu, and Oriya. In [28], a novel 196-element Regional Weighted Run Length (RWRL) feature has been proposed for handwritten Devanagari numerals recognition. The proposed scheme has been evaluated by using SVM classifier. In this work, the authors generated their own samples, which are given by 6000 handwritten Devanagari digit. Recently, an improved handwritten recognition method using a CNN (IHRS-CNN) method has been proposed in [29] for improving the performance of handwritten digit recognition while maintaining the computational complexity. In particular, the IHRS-CNN method makes use of a pure CNN architecture only without the requirement of using any ensemble architecture, which could increase both the cost and computational complexity. The results show that a better performance can be achieved using pure CNN architecture while reducing the computational cost and operational complexity.

Although many deep learning-based classification algorithms have been studied for handwritten digits recognition, the recognition accuracy and the running time remain major issues that need to be addressed. In particular, the current techniques do not provide results that meet the desired accuracy with fast execution time. As such, a careful investigation of a new fast and robust technique is essential to meet the desired accuracy. Furthermore, to date most of the existing research works do not consider the effect of the noise, limiting understanding of their potential application in noise environments. We believe that investigating the robustness of the proposed solution against the noise is crucial to characterize the effectiveness of the features extraction.

To address the aforementioned research challenges, the present paper employs a combination of two orthogonal polynomials—Krawtchouk polynomials (KPs) and Tchebichef polynomials (TPs)—for numeral recognition system. These two well-known orthogonal polynomials are widely used and frequently encountered in the open literature of image representation and signal compression [30,31]. This is due to their powerful capabilities in analyzing the signals’ components and retaining the significant features of the signals quickly and efficiently. In this paper, we adopt these powerful polynomials, more accurately their combination to produce a hybrid orthogonal polynomials, which we call squared Krawtchouk–Tchebichef polynomial (SKTP). In addition, to expedite the process of extracting different types of features (gradient and smooth), the embedded kernel technique is employed. The performance of the proposed approach is evaluated and compared under different numeral recognition datasets. In particular, the proposed method is evaluated under the noise-free environment, which we call it as clean noise. In addition, we investigate the effectiveness of the proposed approach for numeral recognition in the presence of noise distortion. To the best of our knowledge, no previous research works have investigated the combination of two hybrid orthogonal polynomials for handwritten numeral recognition. To this end, we provide the mathematical formulation of the utilized orthogonal polynomials and their moments. Furthermore, we compare the proposed character recognition system with the state-of-the-art recognition methods. The results illustrate that the proposed method achieves almost the highest recognition accuracy in comparison with the state-of-the-art recognition methods in all the scenarios considered. Importantly, the results show that the proposed method is robust against the noise distortion and outperforms the CNN remarkably, which signifies the feasibility and the effectiveness of our proposed method in comparison to the state-of-the-art recognition methods under both clean noise and more practical noise environments.

The paper is organized as follows. In Section 3, the system models of the orthogonal polynomials and orthogonal moment along with their mathematical formulations are introduced. In Section 4, we explain the methodology of the feature extraction process together with the classification process. In Section 5, numerical results are provided in order to characterize the performance of the proposed method and also to validate the effectiveness of the proposed approach in the presence of noise distortion. Finally, the paper is concluded in Section 6.

*Notation*: In this paper, an upper boldface symbol stands for a matrix whereas a lower boldface symbol stands for a vector. The operator transpose is denoted by ${\left(\cdot \right)}^{T}$.

## 3. Mathematical Models of Orthogonal Polynomials and Moments

This section provides the mathematical analysis of the employed orthogonal polynomials and the computation of their moments for two-dimensional signals.

### 3.1. Orthogonal Polynomials

The basic principle of the orthogonal polynomials is to project a signal, which could be a speech or image, in the orthogonal polynomials basis. Orthogonal polynomials have the potential to represent the features of signal in an enhanced, efficient, and non-redundant way. Orthogonal polynomials consist of a square matrix with two axes, where these axes represent the signal index (*x*) and polynomial order (*n*). The elements of the generated matrix are referred to the orthogonal polynomial coefficients. Motivated by the properties of orthogonal polynomials, which allow a combination of any two orthogonal polynomials matrices to generate orthogonal polynomials matrix, this paper explores Tchebichef polynomials and Krawtchouk polynomials and their moments to produce a hybrid form of orthogonal polynomials. This combination results in a squared matrix with orthogonal polynomials termed as SKTP [32], which has unique features combined from both Tchebichef and Krawtchouk polynomials. The hybrid polynomials show a remarkable performance improvement in terms of energy compaction and localization properties in comparison to any sorts of orthogonal polynomials, i.e., Krawtchouk–Tchebichef polynomials only [33]. Such polynomial combinations could also be efficiently applied in communication signal processing to reduce the complexity of RZF and RZFBF precoders [34,35] or to minimize the feedback overhead [36,37]. To this end, this paper proposes an approach based on SKTP aiming to provide a fast handwritten numeral recognition with high accuracy. Accordingly, the *n*-th order of the SKTP, $R_{n}$, can be written as [32]

(1) $$\begin{array}{c} R_{n}\left(x;p\right)=\sum_{i=0}^{N-1}\sum_{j=0}^{N-1}\sum_{l=0}^{N-1}K_{j}\left(i;p\right)\;T_{j}\left(x\right)\;K_{l}\left(n;p\right)\;T_{l}\left(i\right),\\ n,x=0,1,\cdots ,N-1, \end{array}$$

where parameters T and K are the TPs and KPs coefficients, respectively. Furthermore, parameter *p* is the polynomial degree of Krawtchouk polynomial. The *n*-th order normalized Krawtchouk polynomial can be written as [38]

(2) $$K_{n}\left(n;p\right)=\sqrt{\frac{\omega_{K}\left(x\right)}{\rho_{K}\left(n\right)}}\;{}_{2}F_{1}\left(-n,-x;-N+1;\frac{1}{p}\right),$$

where parameters $\omega_{K}$, $\rho_{K}$ represent the weight and norm of the Krawtchouk polynomial, respectively. On the other hand, the *n*-th order normalized Tchebichef polynomial can be expressed as [39]

(3) $$T_{n}\left(x\right)=\sqrt{\frac{\omega_{T}\left(x\right)}{\rho_{T}\left(n\right)}}\;{\left(1-N\right)}_{n}\;{}_{3}F_{2}\left(-n,-x,1+n;1-N;1\right),$$

where parameters $\omega_{K}$$\rho_{K}$ are defined as the weight and norm functions of the Tchebichef polynomial, respectively. The employed SKTP matrix in (1) can be computed by using matrix–matrix multiplication, which preserves the orthogonality due to the unique feature of the orthogonal polynomials. As such, the employed SKTP matrix R can be expressed using a matrix multiplication as

(4) $$R={\left(R_{k}\times R_{t}\right)}^{2},$$

where matrices $R_{k}$ and $R_{t}$ denote Krawtchouk polynomial and Tchebichef polynomial, respectively. It is worth noting that the computation of the Tchebichef polynomial and Krawtchouk polynomial is conducted here using a three-term recurrence algorithm. This is due to the fact that the hypergeometric series of these polynomials (${}_{2}F_{1}$ and ${}_{3}F_{2}$) and the gamma functions are computationally cost, and thus could be infeasible for practical implementation [40]. As such, the three terms recurrence algorithm allows a computational effect solution, which justifies its use here.

### 3.2. Orthogonal Moments

The orthogonal moments (OMs) have recently received considerable attention as key fundamental tools in different digital processing systems [41]. In particular, OMs can be effectively utilized to reduce the noise distortion effect and improve the features representation. The OMs are computationally efficient and have the capability to reduce the numerical error in comparison to the continuous orthogonal moments (COMs). In addition, OMs are scalar quantities that can discover any small changes or distortions that could be introduced or occurred to the signals [42]. Owing to these beneficial capabilities, OMs have been widely used in various signal processing applications. The basic principle of OMs are to project the signal/image on the polynomial basis functions, which could result in scalar quantities that are used in retaining the significant features of the signal/image [40]. This implies that OMs can be considered as shape descriptors (features). OMs can be divided into two types, which are low-order moments (LOMs) and high-order moments (HOMs). The LOMs preserve most of the signal energy, which, in principle, represents the most effective features/information about the signal. On the other hand, the HOMs contain the other details of the signal [43]. In this paper, the moments are computed using the hybrid SKTP. As such, we call the SKTP with the moments combination as a squared Krawtchouk–Tchebichef moments (SKTMs). To this end, for any two-dimensional signal, the SKTM ($M_{nm}$) can be computed as

(5) $$\begin{array}{cc} M_{nm}=\sum_{x=0}^{N_{1}-1} & \sum_{y=0}^{N_{2}-1}R_{n}\left(x;p,N_{1}\right)R_{m}\left(y;p,N_{2}\right)f\left(x,y\right),\\ n=\frac{N_{1}}{2}-1 & ,\frac{N_{1}}{2},\cdots ,\frac{N_{1}-O_{n}}{2},\frac{N_{1}+O_{n}}{2}-1,\\ m=\frac{N_{2}}{2}-1 & ,\frac{N_{2}}{2},\cdots ,\frac{N_{2}-O_{m}}{2},\frac{N_{2}+O_{m}}{2}-1, \end{array}$$

where parameters $O_{n}\;\mathrm{and}\;O_{m}$ represent the maximum order of moments used to represent the two-dimensional signal, and f(x,y) represents the two-dimensional signal/image. Specifically, to achieve a fast computation time and efficient implementation of SKTMs, matrix–matrix multiplication is utilized. Accordingly, the resulting matrix of the moments, M, with *n*th and *m*-th elements, $M_{nm}$, can be expressed as

(6) $$\begin{array}{c} M=R_{1}^{}\times F\times R_{2}^{T}, \end{array}$$

where F denotes the matrix form of the image f(x,y), and $R_{1}\;\mathrm{and}\;R_{2}$ represent the matrix form of orthogonal polynomials with *n*th and *m*-th elements, which are given by $R_{n}\;\mathrm{and}\;R_{m}$. It is noteworthy that the basis functions of orthogonal polynomials can be utilized as an approximate solution for differential equations as discussed in [44].

## 4. The Proposed Methodology for Handwritten Numeral Recognition

In this section, the proposed methodology for handwritten numeral recognition is provided. In particular, to make it simple and easy to clarify, this section is divided into two subsections: the feature extraction process and classification process.

### 4.1. Feature Extraction Process

The feature extraction process is considered as key fundamental part in the recognition system, which is beneficial for signal representation. As such, to enable an efficiently numeral recognition system, a global feature extraction is utilized instead of the local feature extraction. Although an approach based on SKTMs has shown reasonable performance in signal representation [45], this was based on clean noise environment. In particular, the vast majority of research studies on numeral recognition have focused on clean noise environment, thus preventing the characterization of efficient numeral recognition methods in more practical environments. However, when the numeral characters exhibit a noisy environment, numeral recognition would be severely affected, and thus the accuracy of signal/image recognition will be significantly reduced. This paper addresses the aforementioned challenge by taking into account the effect of the noise, which underpins the main contribution of this work. Specifically, this paper applies smoothed and gradient operator [46] to the input image in order to extract the most effective features. To this end, a smoothed kernel is used in this paper to minimize the effect of noise. In addition, this paper exploits the gradient kernel to compute the gradient of the input image for efficient numeral recognition outcome. It is worth pointing out that applying the aforementioned kernels directly is computationally and cost inefficient. Therefore, to increase the computational time, an orthogonal polynomial embedded image kernel technique [46] is explored here. In order to compute the smoothed kernel moments $\Psi_{s}$, the following formula is used,

(7) $$\Psi_{s}=S_{y}\times F\times S_{x},$$

where parameters $S_{x}$ and $S_{y}$ are the SKTP embedded with smoothed operator in the *x* and *y* directions, respectively. To this end, parameters $S_{x}$ and $S_{y}$ can be expressed as [46]

(8) $$\begin{array}{c} S_{x}=R\times H_{sx}, \end{array}$$

(9) $$\begin{array}{c} S_{y}=R\times H_{sy}, \end{array}$$

where matrix R is given in (4), which represents the combination of Krawtchouk and Tchebichef polynomials, and $H_{sx}$ and $H_{sy}$ represent the Toeplitz matrix (An N×N matrix A is a Toeplitz matrix if the i,j elements of A, i.e., ${\left[A\right]}_{i,j}$, satisfies the following ${\left[A\right]}_{i,j}=A_{i+1,j+1}=a_{i-j}.$) matrices of the smoothed operators of vectors $h_{sx}$ and $h_{sy}$, respectively. This paper uses the circularly symmetric complex Gaussian distribution with mean 0 as a smoothing kernel. Accordingly, the smoothing kernel can be expressed as

(10) $$\begin{array}{c} h_{sx}=\frac{1}{2\pi \sigma_{x}^{2}}e^{-\frac{x^{2}}{2\sigma_{x}^{2}}}, \end{array}$$

(11) $$\begin{array}{c} h_{sy}=\frac{1}{2\pi \sigma_{y}^{2}}e^{-\frac{y^{2}}{2\sigma_{y}^{2}}}, \end{array}$$

where $\sigma^{2}$ represents the standard deviation of the Gaussian distribution. The expression in (8) and (9) indicates that no preprocessing operation is required before extracting features.

To compute the moments of gradient image, the SKTMs of the gradient image, which are given in $\Psi_{x}$ and $\Psi_{y}$, can be directly computed from the smoothed moments. Accordingly, the moments of gradient image are obtained as

(12) $$\begin{array}{c} \Psi_{x}=\Psi_{s}\times P_{x}, \end{array}$$

(13) $$\begin{array}{c} \Psi_{y}=P_{y}\times \Psi_{s}, \end{array}$$

where matrices $P_{x}$ and $P_{y}$ can be determined as

(14) $$\begin{array}{c} P_{x}=S_{x}\times G_{x}^{T} \end{array}$$

(15) $$\begin{array}{c} P_{y}=G_{y}\times S_{y}^{T} \end{array}$$

where matrices $G_{x}$ and $G_{y}$ represent the SKTP embedded with gradient operator in the *x* and *y* directions, respectively. Matrices $G_{x}$ and $G_{y}$ can be computed as [46]

(16) $$\begin{array}{c} G_{x}=R\times H_{gx}, \end{array}$$

(17) $$\begin{array}{c} G_{y}=R\times H_{gy}, \end{array}$$

where matrices $H_{gx}$ and $H_{gy}$ represent the Toeplitz matrices of the smoothed operators $h_{gx}$ and $h_{gy}$, respectively. In this paper, a simple gradient operator is exploited as a gradient kernel, which is given as

(18) $$\begin{array}{cc} h_{gx}= & [-1\;\;\;1], \end{array}$$

(19) $$\begin{array}{cc} h_{gy}= & {\left[-1\;\;\;1\right]}^{T}. \end{array}$$

After the moments of the smoothed and gradient are numerically obtained, they can be concatenated in order to form a unique feature vector (FV). The obtained feature vector is then normalized to ensure similar dynamic range [38,47]. The normalization process is used because the values of features lie within wide ranges; thus, the effect of large features values dominate small features values [40].

**Figure 1:**
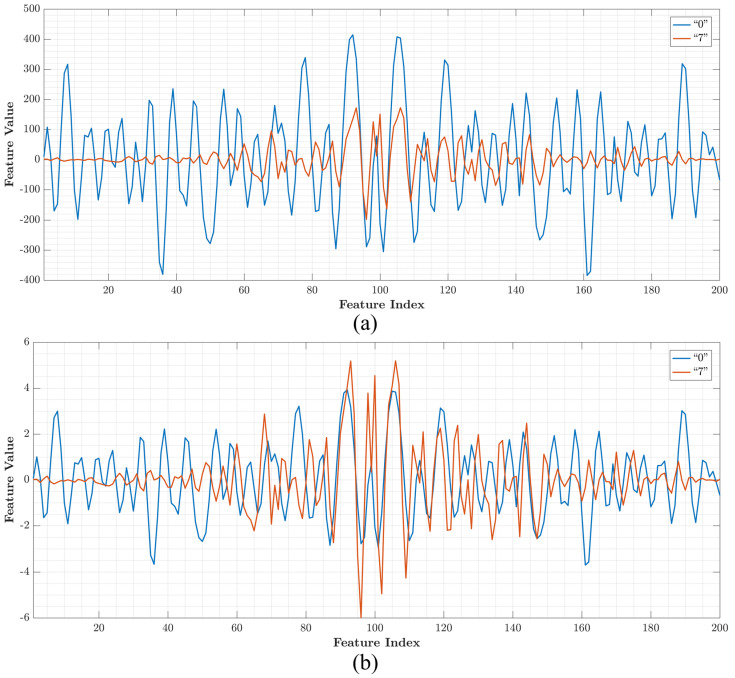
Plot of feature vector samples with indices from 1 to 200 (**a**) without normalization and (**b**) with normalization.

Figure 1a shows samples of feature vectors without normalization and Figure 1b shows samples of features with normalization using zero mean and standard deviation of 1. Unlike the normalize feature vectors, the feature vectors without normalization show values with a wide range [−400, 600].

**Figure 2:**
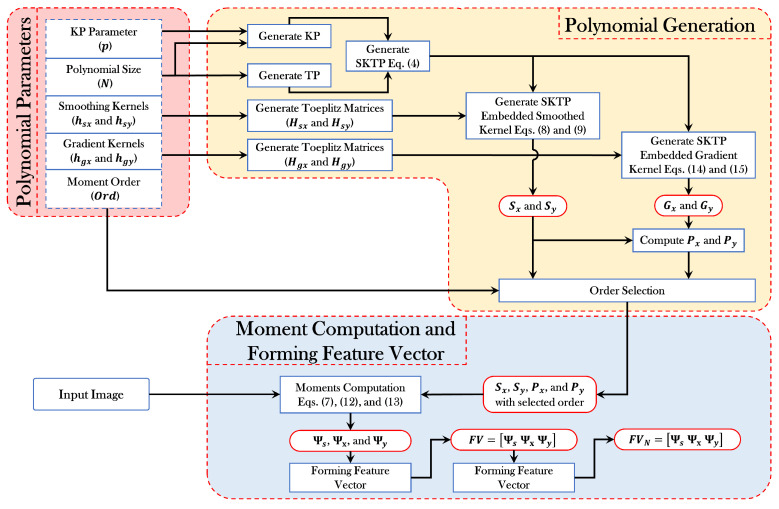
A flow chart of the feature extraction process.

In order to make the proposed methodology of feature extraction more clearly, the block diagram of the feature extraction process is provided in Figure 2.

### 4.2. Classification Process

After obtaining the normalized feature vector, an identified number value (ID) for each input image is obtained based on a classifier. Note that the feature vector is considered as an input to the classifier. A method using SVM is utilized in this paper to accomplish the classification process. The reason behind selecting the SVM method is due to its capability to maximize the margin between separation classes of the hyperplane and data, which is achieved by generating a hyperplane [48,49,50]. This SVM can also minimize the structural risk by controlling the out-of-sample error [48,49]. In addition, SVM is more adequate for recognition as it is more resistant to the noisy environment [49]. By generating a hyperplane, SVM can separate the positive and negative images [50]. The LIB-SVM with kernel function is used for SVM implementation [47,51].

**Figure 3:**
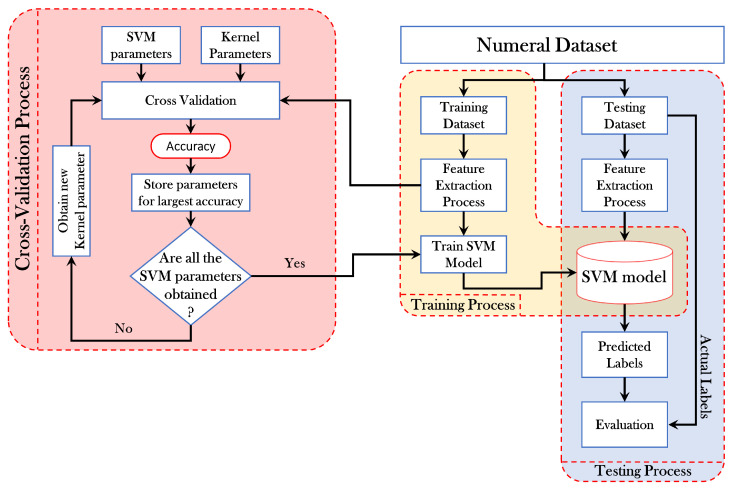
A schematic diagram for support vector machine (SVM) training and testing process.

Figure 3 shows a schematic diagram of the classification process using the SVM method, which includes training and testing process. The SVM kernels utilize polynomial and radial basis function (RBF). These choices are considered as an effective classification mechanism since these kernels show nonlinear separation between classes [47]. To ensure high prediction accuracy, the cross-validation process is carried out. This allows the best kernel parameters to be obtained. Note that the cost and gamma are essential parameters that are required to be tuned. To this end, five-fold cross-validation is applied. The ranges of the parameters for cost and gamma are considered to be ($2^{0},2^{1},\ldots ,2^{5}$) and ($2^{-10},2^{-9},\ldots ,2^{0}$). The cost and gamma parameters show high accuracy on the testing set.

## 5. Experimental Results and Discussion

This section presents several simulation results, which characterize the performance of the proposed numeral recognition in different datasets. We first evaluate different choices of kernels in order to select the best SVM kernel. Furthermore, we compare the proposed numeral recognition method with the state-of-the-art recognition methods. The following subsection describes the datasets used in this paper.

### 5.1. Database Description

**Figure 4:**
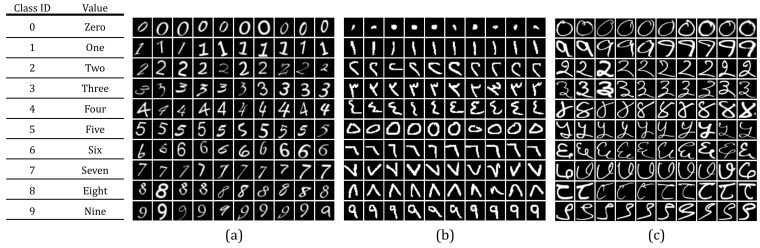
Samples from dataset images (**a**) Roman, (**b**) Arabic, and (**c**) Devanagari.

In this paper, several benchmarks handwritten numeral datasets are used in the experiments. These datasets include Roman, Devanagari, and Arabic scripts. Among the diverse set of datasets, Roman, which originally came from the Greek alphabet [6], seems to be the most popular handwritten numeral. This is mainly due to the fact that Roman is considered as a second language in the vast majority of countries around the world. Arabic handwritten numerals are widely used in the Middle East countries as well as part of Asia such as in India. On the other hand, Devanagari script is also considered in this paper, which is not only the most popular script used for the Hindi language, but also used by more than 120 different languages around the world [6]. This can justify our focus on these particularly essential handwritten numerals in our experiments. To this end, the MINST numeral dataset provided in [12] is used in this paper. The MINST includes 10 numerals, i.e., 10 classes with 1000 images for each class. The Arabic numeral dataset (CMATERdb 3.3.1) is obtained from the work in [52], wherein over 10,000 images are explored with over 1000 images for each class. Finally, we obtain the dataset of Devanagari (CMATERdb 3.2.1) from the work in [52]. In the Devanagari case, over 20,000 images are used with about 2000 images for each numeral. Figure 4 elaborates different samples for different datasets, which include Roman, Arabic, and Devanagari. The following subsection evaluates the performance of the proposed numeral recognition method based on the above discussed datasets.

### 5.2. Characterizing the Performance of the Proposed Numeral Recognition Using Different Kernels Methods

The main objective of this paper is to identify the best handwritten numeral recognition that achieves the best accuracy. To this end, for a given input image, we identity the best recognition from different types of numerals. The feature extraction process and the training and testing process provided in Figure 2 and Figure 3, respectively, and discussed in detail in Section 4, are exploited here for numeral recognition. In particular, for each dataset (e.g., Roman, Arabic, and Devanagari), the samples are divided equally into two parts, i.e., one for the training phase and the other part for testing phase (see Table 1 for details). For each phase, the feature extraction process is shown in Figure 2, where this process is utilized to represent each image by its related features.

The proposed numeral recognition method is implemented based on two SVM kernels, which are defined as the polynomial and radial basis function (RBF) kernels. For the radial basis function kernel, the tuned parameters are given by the cost (*C*) and gamma (γ). On the other hand, for the polynomial kernel, the parameters are given by the cost (*C*), gamma (γ), and the kernel coefficient (coef0). More details about the polynomial as well as radial basis function kernels can be found in [51]. The obtained kernels coefficients are shown in Table 2.

**Figure 5:**
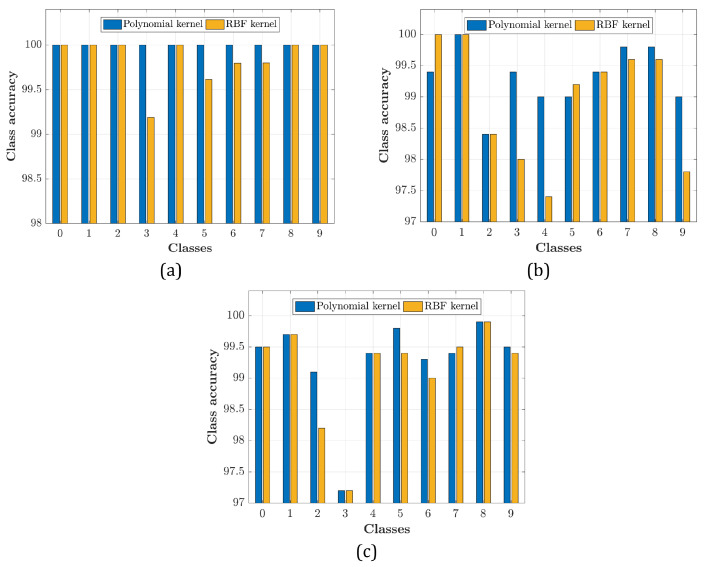
Comparison of classes accuracy using polynomial and RBF kernels for (**a**) Roman, (**b**) Arabic, and (**c**) Devanagari.

**Figure 6:**
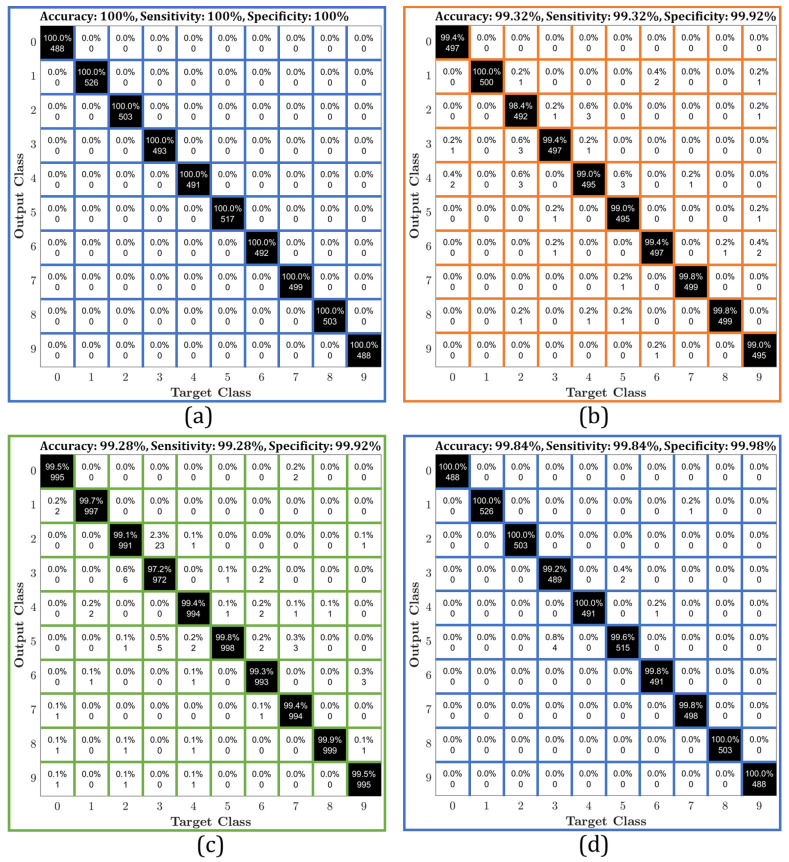

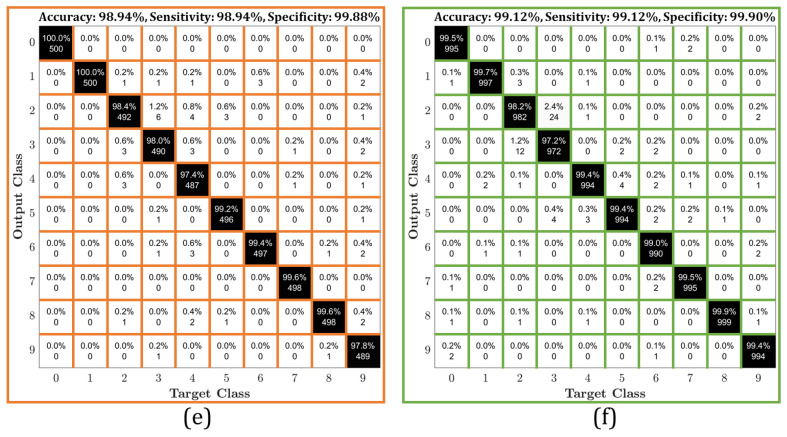
Confusion matrix of the numeral recognition system using (**a**) polynomial kernel for Roman dataset, (**b**) polynomial kernel for Arabic dataset, (**c**) polynomial kernel for Devanagari dataset, (**d**) RBF kernel for Roman dataset, (**e**) RBF kernel for Arabic dataset, and (**f**) RBF kernel for Devanagari dataset.

Figure 2 depicts that the polynomial kernel using the SVM method achieves better overall preference in terms of accuracy than the RBF kernel. As such, the polynomial kernel can be considered as a more robust recognition technique in comparison to the RBF kernel. For more clarification about the differences between polynomial and RBF kernels, we have provided Figure 5. In particular, Figure 5 shows the accuracy comparison between polynomial and RBF based on Roman, Arabic, and Devanagari datasets considered. From Figure 5a, we observe that for classes 3, 5, 7, and 8, the RBF kernel is less accurate than the polynomial kernel when the Roman dataset is used. For the Arabic dataset in Figure 5b, however, the class accuracy for polynomial kernel is slightly degraded in comparison to Figure 5a. However, the results in Figure 5b illustrate that the RBF kernel is considerably degraded when Arabic dataset is used, especially for classes 3, 4, and 9. Overall, the results show that the polynomial kernel is more accurate than the RBF kernel for almost all the classes considered when Arabic dataset is used. On the other hand, Figure 5c investigates the accuracy of polynomial and RBF kernel based on the Devanagari dataset. The results show that for Devanagari dataset, again, the RBF kernel is less robust in recognition than the polynomial kernel where the accuracy has been considerably dropped, especially when we considered classes 2, 5, 6, and 9. For more elucidation, the confusion matrices polynomial and RBF kernels are evaluated based on all the datasets. To achieve this purpose, Figure 6 conducts a comparison of the accuracy between the polynomial and RBF kernels.

Specifically, Figure 6a–c provides a confusion matrix of the numeral recognition using polynomial kernel based on Roman, Arabica, and Devanagari, respectively. Figure 6d–f illustrates the results of the confusion matrix of the numeral recognition using RBF kernel based on Roman, Arabica, and Devanagari, respectively. Figure 6 demonstrates clearly that, based on the confusion matrix of the numeral recognition, the polynomial kernel outperforms RBF kernel in all datasets considered. Based on the experiments above, and due to the accuracy that polynomial kernel provides over a wide range of comparisons, we consider the polynomial kernel with SVM method for feature extraction in the rest of the paper. In particular, polynomial kernel is used here with SVM in the classification process for numeral recognition system and through the comparison with the state-of-the-art methods.

### 5.3. Comparing Performances of the Proposed Method and the Existing State-of-the-Art Methods for Numeral Recognition

To evaluate the performance of the proposed algorithm, different comparisons with existing recognition methods are carried out in this subsection. Unless otherwise specified, the comparisons are conducted based on clean noise environment, i.e., without introducing the noise. Our proposed method use SVM as a classifier with polynomial kernel. Table 3 provides a comparison based on the Roman numeral recognition. Different recognition methods, which use different classifier types, are compared with each other and with our proposed method. To this end, a handwritten recognition based on moments fusion (MF) [6] is used. MF recognition method uses two different classifier types, which are defined as a multilayer perceptron (MLP) and SVM. Conventional neural network CNN, which uses 5-layers CNN, has been proposed in [27]. We consider this 5-layers CNN method in the comparison and we term it CNN-5 for brevity. We also compare our method with the statistical–topological feature combination (STFC) proposed in [26]. The fourth recognition method that we consider in the comparison is IHRS-CNN. As such, the classifier type for the CNN-5 and IHRS-CNN methods is CNN.

The results in Table 3 show that the STFC and MF methods that use SVM classifier achieve the lowest accuracy recognition levels, which are below 99%. On the other hand, CNN-5-based CNN classifier achieves 99.10% recognition accuracy, while the MF-based MLP classifier and IHRS-based CNN classifier achieve the same recognition accuracy of 99.77%. Interestingly, the proposed method achieves the highest accuracy level, i.e., 100%, among all the methods considered. This signifies the feasibly of our proposed method in comparison with other methods used for handwritten Roman numerals recognition. Particularly, the proposed method provides an accuracy improvement of 0.23% in comparison with the MF-based MLP and IHRS methods, and 1.25% improvement in comparison with MF-based SVM.

Having demonstrated the feasibility of the proposed method with the Roman numeral recognition, it is pertinent to compare the proposed algorithm performance recognition with that obtained from the conventional recognition methods based on Arabic numeral recognition. To this end, the proposed algorithm is compared with several different algorithms. For example, shape features (SF) [25] using random forest and MLP for classification are considered in the comparison based on Arabic numeral recognition. In addition, convolutional neural network (CNN), which is proposed in [19] for Arabic handwritten numerals, is also used here in the comparison. The rest of the methods that are used in the comparison are similar to those already considered with the Roman numeral recognition, which are MF-based MLP and SVM classifiers [6] and STFC with SVM classifier [26]. Table 4 provides an accuracy comparison of the aforementioned methods with our proposed method. The results show that CNN [19] method based on CNN classifier achieves the lowest accuracy recognition level, which is below 97.40%. The results demonstrate that the proposed numeral recognition algorithm outperform the existing algorithms for handwritten Arabic numeral recognition, which achieves 99.32% recognition accuracy.

So far, the results presented are based on the Roman and Arabic dataset, in what follows we compare the proposed method with the state-of-the-art methods based on Devanagari handwritten numeral. To this end, a regional weighted run length feature (RWRLF) [28] algorithm, wherein the SVM and MLP classifier are employed for classification, is used here for comparison. The rest of the methods that are compared here are similar to those already used with the Roman and Arabic numeral recognition, which are MF-based MLP and SVM classifiers [6], SF [25] with random forest and MLP classifier, and STFC with SVM classifier [26]. Table 5 presents the accuracy comparison of different recognition with their corresponding classifiers. The results show that the MF based on MLP classifier method achieves the best accuracy performance of 99.30%. However, the proposed algorithm still achieves a comparable results to that obtained based on the MF method with only 0.02% accuracy difference that is considered negligible. Nevertheless, our proposed method outperforms the MF method in both Roman and Arabic handwritten numeral recognition. Furthermore, the proposed method considerably outperforms all the rest of the methods considered in the comparison for the Devanagari handwritten numeral. This again signifies the feasibility of our method in comparison to the other methods considered.

From the previous comparison, the proposed method seems to be almost outperforming all the existing algorithms based on the clean environment. To illustrate the effectiveness and the robustness of our proposed algorithm, different noisy environments are also considered. We select the updated state-of-the-art algorithm, which is IHRS-CNN [29]. The implementation of IHRS-CNN algorithm is also straightforward. This can justify its use in the comparison with different noisy environments. The noisy environments are chosen to be Gaussian noise with two different variance values, i.e., ($\sigma^{2}=0.01$ and $\sigma^{2}=0.05$). Moreover, the Salt and Pepper noise with two different density values, i.e., d=0.01 and d=0.05 are also considered. Finally, we use the blur noise in the comparison, which is implemented using an averaging filter with size of 3×3. In addition, as in previously presented results, the comparison is conducted based on the Roman, Arabic, and Devanagari numerals. The results of the comparison are provided in Table 6.

As illustrated in Table 6, for the Roman numerals, the proposed algorithm shows a stable recognition accuracy in clean and noisy environments. However, there is a slight degradation in the recognition accuracy when the standard deviation of the Gaussian noise is increased, i.e., $\sigma^{2}$ = 0.05. On the other hand, the recognition accuracy performance of the IHRS-CNN method degrades in all the noisy environments considered. For example, the IHRS-CNN shows 2.7% and 9.8% for the Gaussian noise with $\sigma^{2}$ = 0.01 and $\sigma^{2}$ = 0.05, respectively. For the Arabic numerals, the proposed method achieves almost the same accuracy of about 99% in all the noisy environments considered. Clearly, the results show that the proposed method again is more accurate than the state-of-the-art method in all cases. In particular, the proposed method achieves a maximum gain in the accuracy of 5.28% in the Salt and Pepper noisy environment with density d=0.05. For the Devanagari numeral recognition, the proposed method again considerably outperforms the IHRS-CNN method. Here, the proposed method achieves a maximum gain of 2.97 over the IHRS-CNN method in the Gaussian noise with standard deviation of $\sigma^{2}$ = 0.05.

In terms of execution time, the proposed method with Roman and Arabic numerals are executed with 24.2 and 15.1 s, respectively, while the CNN spends 26.7 and 17.9 s, respectively, to get executed. Note that the time is measured for the entire test set for each type of numerals. This clearly implies that the proposed method has nearly comparable results with the existing methods in terms of execution time. Overall, the results presented show that the proposed algorithm is still robust in the noisy environments in comparison to the state-of-the-art IHRS-CNN method.

## 6. Conclusions

This paper presented a new codesign of orthogonal polynomials with their associated moments to improve handwritten numeral recognition accuracy. The proposed algorithm have been evaluated for three different numeral recognitions: Roman, Arabic, and Devanagari. The results demonstrated that the proposed approach achieves the highest recognition accuracy in comparison to the state-of-the-art numeral recognition methods. Importantly, the numerical results showed that the proposed approach is robust against the noise distortion, which signifies the effectiveness of the proposed approach under realistic environments.

## Figures and Tables

**Table 1:** Size of the sample for each dataset, and the size of the training and testing sets.

Dataset	Sample Size	The Size of the Training Set	The Size of the Testing Set
Roman	10,000	5000 (50%)	5000 (50%)
Arabic	10,000	5000 (50%)	5000 (50%)
Devanagari	20,000	10,000 (50%)	10,000 (50%)

**Table 2:** Comparing the polynomial and RBF kernels coefficients based on the SVM method for Roman, Arabic, and Devanagari datasets.

Dataset	Kernel	*C*	γ	coef0	Degree	Accuracy
Roman	RBF	$2^{6}$	$2^{-9.6}$	-	-	99.80
	Polynomial	$2^{6}$	$2^{-7.2}$	0	4	100
Arabic	RBF	$2^{6}$	$2^{-9.6}$	-	-	98.94
	Polynomial	$2^{6}$	$2^{-9.6}$	−0.15	4	99.32
Devanagari	RBF	$2^{6}$	$2^{-9.6}$	-	-	99.12
	Polynomial	$2^{6}$	$2^{-9.6}$	−0.16	4	99.28

**Table 3:** Comparison between the proposed method and the state-of-the-art methods based on Roman numeral recognition in clean environment.

Method	Classifier Type	Dataset	Accuracy %
MF [6]	MLP	MNIST	99.77
MF [6]	SVM	MNIST	98.75
STFC [26]	SVM	MNIST	98.90
CNN-5 [27]	CNN	MNIST	99.10
IHRS-CNN [29]	CNN	MNIST	99.77
Proposed	SVM	MNIST	100.00

**Table 4:** Comparison between the proposed method and the state-of-the-art methods for Arabic numeral recognition in clean environment.

Method	Classifier Type	Dataset	Accuracy %
SF [25]	random forest	CMATERDB 3.3.1	98.40
SF [25]	MLP	CMATERDB 3.3.1	98.20
MF [6]	MLP	CMATERDB 3.3.1	98.92
MF [6]	SVM	CMATERDB 3.3.1	97.95
STFC [26]	SVM	CMATERDB 3.3.1	98.40
IHRS-CNN [29]	CNN	CMATERDB 3.2.1	98.42
CNN [19]	CNN	CMATERDB 3.3.1	97.40
Proposed	SVM	CMATERDB 3.3.1	99.32

**Table 5:** Comparison between the proposed method and the state-of-the-art methods for Devanagari numeral recognition in clean environment.

Method	Classifier Type	Dataset	Accuracy %
SF [25]	Random Forest	CMATERDB 3.2.1	98.01
SF [25]	MLP	CMATERDB 3.2.1	97.40
MF [6]	MLP	CMATERDB 3.2.1	99.30
MF [6]	SVM	CMATERDB 3.2.1	97.98
RWRLF [28]	SVM	CMATERDB 3.2.1	95.03
RWRLF [28]	MLP	CMATERDB 3.2.1	94.47
STFC [26]	SVM	CMATERDB 3.2.1	98.70
IHRS-CNN [29]	CNN	CMATERDB 3.2.1	97.60
Proposed	SVM	CMATERDB 3.2.1	99.28

**Table 6:** Comparison between the proposed method and IHRS-CNN [29] method for Roman, Arabic, and Devanagari numeral recognition in both clean and noisy environments.

Roman			Arabic			Devanagari		
Environment	Proposed	IHRS-CNN [29]	Environment	Proposed	IHRS-CNN [29]	Environment	Proposed	IHRS-CNN [29]
Clean	100.00	99.77	Clean	99.32	98.42	Clean	99.23	97.6
Gaussian noise $\sigma^{2}=0.01$	100.00	97.08	Gaussian noise $\sigma^{2}=0.01$	99.12	97.78	Gaussian noise $\sigma^{2}=0.01$	99.17	97.32
Gaussian noise $\sigma^{2}=0.05$	96.68	90.00	Gaussian noise $\sigma^{2}=0.05$	99.00	94.76	Gaussian noise $\sigma^{2}=0.05$	98.66	95.69
Salt & Pepper noise (d = 0.01)	100.00	93.68	Salt & Pepper noise (d = 0.01)	99.08	97.68	Salt & Pepper noise (d = 0.01)	99.22	97.4
Salt & Pepper noise (d = 0.05)	99.90	90.76	Salt & Pepper noise (d = 0.05)	99.12	93.84	Salt & Pepper noise (d = 0.05)	99.14	96.69
Blur (filter size = 3×3)	100.00	96.88	Blur (filter size = 3×3)	99.02	98.08	Blur (filter size = 3×3)	99.23	97.27

## Data Availability

The data presented in this study are available on request from the corresponding author.

## Abbreviations

The following abbreviations are used in this manuscript:

OCR	optical character recognition
SVM	support vector machine
NIST	national institute of standards and technology
MNIST	modified NIST
CMATER	Center for Microprocessor Applications for Training Education and Research
CNN	convolutional neural network
HOPP	Histogram of Oriented Pixel Positions
PLSS	Point-Light Source-based Shadow
KP	Krawtchouk polynomials
TP	Tchebichef polynomials
SKTP	squared Krawtchouk–Tchebichef polynomial
COM	continuous orthogonal moment
OM	orthogonal moment
LOM	low-order moments
HOM	high-order moments
SKTM	squared Krawtchouk–Tchebichef moment
FV	feature vector
